# The 9p21 Locus Is Associated with Coronary Artery Disease and Cardiovascular Events in the Presence (but Not in the Absence) of Coronary Calcification

**DOI:** 10.1371/journal.pone.0094823

**Published:** 2014-04-14

**Authors:** Ling Gong, Jinxing Chen, Jinguo Lu, Lizi Fan, Jinghan Huang, Yu Zhang, Bin Lv, Rutai Hui, Yibo Wang

**Affiliations:** 1 State Key Laboratory of Cardiovascular Disease, Sino-German Laboratory for Molecular Medicine, Fuwai Hospital, National Center for Cardiovascular Diseases, Chinese Academy of Medical Sciences and Peking Union Medical College, Beijing, China; 2 Department of Radiology, Fuwai Hospital, National Center for Cardiovascular Diseases, Chinese Academy of Medical Sciences and Peking Union Medical College, Beijing, China; 3 Departement of Medical Ultrasonic, Fuwai Hospital, National Center for Cardiovascular Diseases, Chinese Academy of Medical Sciences and Peking Union Medical College, Beijing, China; 4 Heart Function Testing Center, Fuwai Hospital, National Center for Cardiovascular Diseases, Chinese Academy of Medical Sciences and Peking Union Medical College, Beijing, China; Children's National Medical Center, Washington, United States of America

## Abstract

Variants at the 9p21 locus have been associated with coronary artery disease (CAD); coronary artery calcification (CAC) is related to CAD and other cardiovascular events. To determine the association of the 9p21 locus with CAD in the presence and absence of CAC, 4 groups were enrolled in a case-control study, including 527 CAD patients without CAC, 692 CAD patients with CAC, 585 individuals with simple CAC but no CAD, and 725 healthy controls. The rs1333049 representing the locus was associated with CAD in the presence of CAC (odds ratio = 1.38 in allelic analysis, 95%CI, 1.19–1.60, *P*<0.001), but not in the absence of CAC. Additionally, rs1333049 was not associated with simple CAC or CAC severity/extent in CAD patients with CAC. 849 CAD patients undergoing revascularization (660 with CAC and 189 without CAC) were enrolled in a cohort study to test its association with cardiovascular events in CAD patients with and without CAC in a 3-year follow-up. rs1333049 was significantly associated with the incidence of cardiovascular events in non-target vessels in patients with CAC (hazard ratio = 1.44, 95%CI, 1.08–1.91, *P* = 0.012), but not in those without CAC. The variants at the 9p21 locus were related to CAD and post-revascularization events only in the presence of CAC, suggesting that they may confer risk of calcification-related coronary atherosclerosis.

## Introduction

Genome-wide association studies (GWAS) have revealed a highly significant association between the variation on chromosome 9p21 and the risk of coronary artery disease (CAD)[1]–[4], which has been validated by studies on different racial and geographic subgroups, independent of traditional risk factors [5]. However, the molecular basis underlying this relationship has remains unknown.

CAD is a chronic multistage inflammatory disease which can progress to acute coronary syndrome (including myocardial infarction and unstable angina) and sudden cardiac death. Multiple cellular pathways in different tissues contribute to the pathogenic processes resulting in CAD. Genetic factors can affect CAD by acting at different stages in its clinical evolution, such as in plaque rupture or the subsequent thrombosis resulting in an increase in the risk of myocardial infarction, and coronary atherosclerosis, resulting in CAD.

Variants of the 9p21 locus have been associated with coronary artery calcification (CAC), premature atherosclerosis, and cardiovascular events (CV events), such as myocardial infarction (based on studies in multiple ethnic groups) [5]–[13]. Additional evidence supports the association of variants on 9p21 with abdominal aortic aneurysms and larger abdominal aorta diameters, therefore affecting vascular structure, while sparing an effect on traditional cardiovascular disease risk factors [9], [13], [14]. CAC, which is associated with a change in vascular structure, can be observed in patients with coronary atherosclerosis (intimal calcification) and in chronic kidney disease, diabetes, and aging (artery tunica media calcification). It has been proven that active inflammatory responses (by macrophages and basic calcium phosphate crystals) are related to atherosclerosis and pathogenesis. Vascular calcifications are the result of an active and inflammatory regulated process; subpopulations of vascular cells are susceptible to inflammatory factors, such as cytokines, oxidized lipids, and monocyte-macrophage products, which promote osteogenesis and matrix calcification in vascular cell culture (cell calcification). Near-infrared fluorescence imaging has found that atherosclerotic mineralization is linked with inflammation at its earliest stages [15].

The conserved sequence within the 9p21 locus has functional enhancer activity which controls the expression of nearby genes modulating inflammation and cell proliferation, therefore promoting atherosclerosis and CAC [16], [17]. In genetic association studies, it has become common to use intermediate phenotypes as outcomes because the power to detect associations can be enhanced by reducing the number of genes potentially responsible for the phenotype, thereby the fraction of the variance explained by any single factor or gene will be increased. In addition, intermediate traits display less heterogeneity and are therefore much easier to define than clinical disease. In this study, we first tested the association of the 9p21 locus represented by rs1333049 with CAD in the presence and absence of coronary calcification in a case-control study, and then assessed its relationship with CV events in a cohort study.

## Material and Methods

Previous GWAS have demonstrated that rs1333049, which is most strongly associated with CAD and its major complication myocardial infarction, is the lead variant at the 9p21 locus [4], [5]. rs1333049, representing the 9p21 locus, was genotyped in our case-control study and cohort study. The primary populations were used in our previous study [18]. All subjects self-reported as Han nationality living in northern China.

### Ethics Statement

All subjects provided written informed consent. The study protocol was conducted in accordance with the Declaration of Helsinki Principles (revised in 1983), and was reviewed and approved by the Ethics Committee of Fuwai Hospital, National Center for Cardiovascular Diseases, Chinese Academy of Medical Sciences, Beijing, China.

### Subjects for the case-control study and determination of the CAD subtypes

Subjects in the first study were enrolled from an outpatient cohort, aged 45–70 years, who underwent coronary computed tomography angiography (CCTA) in Fuwai Hospital for CAD from April 2007 to December 2010. The main complaints and symptoms of these patients included angina, atypical chest pain, and chest distress with multiple risk factors. CCTA was performed with the use of a 64-slice multidetector scanner as previously described [19]. CAD was diagnosed as ≥50% narrowing of the lumina of at least 1 major coronary artery. CAC was quantified using the Agatston score method, incorporating both density and area. We defined presence of calcification as a CAC score>0, absence of calcification as a CAC score = 0, and simple CAC as calcification on the arterial wall but without non-calcified components. The sum of the scores in the left main, left anterior descending, circumflex, and right coronary arteries were considered the CAC score. Patients with large scale calcification which confused the determination of non-calcified component were excluded. The results of CCTA were independently reviewed by two radiologists blinded to randomized allocation. Patients with prior CV events who did not show narrowing of the lumina, patients with previous CAD diagnosed by CCTA or angiography, and patients undergoing revascularization were excluded. The subjects without CAC or narrowing of coronary lumen determined by CCTA were enrolled as controls. Patients with hematological diseases, peptic ulcers, liver and kidney dysfunctions, infections, autoimmune diseases, and tumors were also excluded in this study. We also noted the age, sex, indication of CCTA, and diagnostic test results before imaging based on the patients' files.

### Cohort study with patients undergoing revascularization

Patients treated with coronary artery bypass grafting surgery (CABG) or drug-eluting stents (DES) at Fuwai Hospital were enrolled from April 2006 to December 2006. Patients with previous revascularization or other cardiac surgery, liver disease, or malignant tumor were excluded from the study. The inclusion criteria also included regular antiplatelet therapy, aspirin (100 mg) plus clopidogrel (75 mg) daily post-operation; other antiplatelet therapy or warfarin users were excluded. CV events were defined as reinfarction, coronary revascularization, and cardiovascular death. The follow-up started from the date of revascularization until the date of the first CV events, or the end of the 3 year follow-up period. Major events were recorded based on hospital records, direct telephone contact, and computer records. Supplemental information was obtained from the patient's family and their physicians. In contrast to the results published by Hoppmann et al. in 2009 [20], Ardissino et al. in 2011 hypothesized that a positive association of the locus was found with CV events and the progression of atherosclerosis due to the elimination of interference caused by coronary revascularization [21]. Events occurring in target vessels and in non-target vessels were also recorded (the non-target vessel refers to the vessel which has not previously been revascularized).

### Biological variable determination and clinical data collection

Blood samples were collected after a 12-hour fast before cardiovascular procedures. The plasma and cell buffy coat were kept at −70°C. Genomic DNA was extracted and biological variables were determined within 3 months. A complete clinical history was obtained from all subjects. In addition to neurological history and family history of hypertension, CAD, and diabetes mellitus (DM), the following vascular risk factors were also recorded: history of vascular disease, cigarette smoking, body mass index (BMI), systolic blood pressure (SBP), diastolic blood pressure (DBP), blood glucose, high density lipoprotein-cholesterol (HDL-C), total plasma cholesterol (TC), and triglycerides (TG). Plasma biochemical parameters were assayed by an automatic analyzer (Hitachi 7060, Hitachi, Japan). LDL-C was calculated using the Friedewald formula. Hypertension was based on 3 independent measures of blood pressure >140/90 mmHg or the use of antihypertensive drugs. DM was diagnosed if the subject had a fasting glucose >7.0 mmol/L or >11.1 mmol/L at 2 hours after oral glucose challenge, or both. All lipid measurements were determined by a CDC qualified laboratory at Fuwai hospital.

### Genotype analysis

A DNA isolation kit, RelaxGene Blood DNA System (Tiangen, Beijing, China), was used for preparing genomic DNA following the recommendations of the manufacturer. Genotyping was performed by using MassARRAY high-throughput DNA analysis with matrix-assisted laser desorption/ionization time-of-flight mass spectrometry (Sequenom, Inc., San Diego, CA, USA). The primers were designed by MassARRAY Assay Design software (Version 3.1). rs1333049 was genotyped using iPLEX Gold technology (Sequenom) followed by an automated data analysis with the TYPER RT software Version 4.0. Reproducibility of genotyping was confirmed by bidirectional sequencing in random 10% population, and the reproducibility was 99.1%.

### Statistical analysis

Quantitative variables were compared with the one-way ANOVA test. A chi-square test was used to test for qualitative variables, genotype/allele frequencies, and the Hardy-Weinberg equilibrium of the variants. The effect of rs1333049 on CAD was expressed as odds ratio. Relative risk analysis was carried out with 2×2 cross tabulation (crude OR) and logistic regression (adjusted OR) for the adjustment of conventional risk factors. A linear regression model was used to assess the correlation between rs1333049 and CAC extent/severity and CAD extent, ln(CAC score+1), the number of calcified arteries was as the dependent variable; all analyses were performed under an additive genetic model. Cox proportional-hazards models were used to examine the association between rs1333049 and CV event incidence after adjustment for age, gender, and other covariates, in which the allele coding was defined as the number of minor alleles. Analyses were performed using SPSS 13.0 for Windows.

The statistical power of our study was estimated by G*power and SAS programs. In the case-control study, minor allele frequency was 0.468 and 0.522 in controls and CAD cases, respectively. Our study could reach >85% power. In the cohort study, our sample size could also get >85% power according to the frequency of events in non-target vessels.

## Results

### rs1333049 was associated with CAD in the presence of CAC, but not with CAD in the absence of CAC or simple CAC

The clinical characteristics of the case-control study population are shown in Table 1. The rs1333049 genotype distribution fulfilled expectations of Hardy-Weinberg equilibrium in all groups. The associations of rs1333049 with each sub-phenotype in subgroups are shown in Table 2. A strong association of risk allele was identified only in CAD in the presence of CAC, but not in CAD in the absence of CAC or in simple CAC. The significant association remained after adjustment with conventional risk factors.

**Table 1:** Clinical characteristics of subjects in the case-control study.

	Control	Simple CAC	CAD without CAC	CAD with CAC
Number	725	585	527	692
Age, y	54.9(7.6)	56.8(8.4)†	55.7(8.3)	54.4(6.9)
Men, %	70.6	70.4	70.8	71.0
SBP, mmHg	123.8(13.6)	126.3(13.0)†	124.3(14.7)	123.5(12.6)
DBP, mmHg	77.9(9.2)	79.2(11.1)*	78.4(9.6)	78.7(9.1)*
BMI, kg/m^2^	25.2(3.4)	26.0(3.6)†	25.7(3.5)†	25.9(3.2)†
TG, mmol/L	1.76(8.81)	1.89(8.10)*	1.74(10.80)	1.79(10.10)
TC, mmol/L	5.01(1.20)	5.34(1.06)†	4.79(1.31)†	4.80(1.30)†
HDL-C, mmol/L	1.22(0.49)	1.28(0.30)†	1.10(0.38)†	1.12(0.39)†
LDL-C, mmol/L	2.72(0.83)	3.02(1.03)†	2.52(0.88)†	2.64(0.93)†
Glucose, mmol/L	6.01(1.66)	6.05(1.42)	6.02(1.67)	6.11(1.75)
Cigarette smoking, %	45.0	56.6†	61.3†	66.0†
Hypertension history, %	53.2	56.8	56.5*	64.7†
DM history, %	28.0	35.4†	36.4†	39.2†
ln(CAC score+1)		3.09(1.74)		4.64(1.87)

Age, BMI, SBP and DBP, glucose, HDL-C, LDL-C, and TC values are given as mean (SD); TG values as median (range), and other values as percentages.

*P<0.05, †*P*<0.01 *vs.* control.

**Table 2:** The distribution of rs1333049 in the case-control study.

	Genotype n (%)			Odds Ratio (95% CI)		
	GG	GC	CC	Allele C vs. G	Genotype in Dominant Model	Genotype in Recessive Model
Control	207(28.6)	358(49.4)	160(22.1)			
Simple CAC	161(27.5)	306(52.3)	118(20.2)	0.98(0.84–1.15)	1.03(0.79–1.34)	0.91(0.66–1.25)
CAD without CAC	146(27.7)	248(47.1)	133(25.2)	1.08(0.92–1.27)	1.04(0.75–1.36)	1.14(0.88–1.48)
CAD with CAC	145(21.0)	335(48.4)	212(30.6)	1.38(1.19–1.60)‡	1.49(1.17–1.90)†	1.54(1.21–1.97)‡

Odd ratios in allelic analysis were crude OR, while odds ratios in genotype analysis were adjusted OR stratified by age, sex, BMI, SBP, DBP, cigarette smoking, glucose, HDL-C, LDL-C, TC, TG, hypertension, and DM status.

† *P*<0.01, ‡*P*<0.001, control group as reference.

### No association was observed between rs1333049 and the number of affected coronary arteries

In CAD patients, we analyzed the association between rs1333049 and the number of affected coronary arteries (defined as main stenosis ≥50%). No association was found in CAD patients. When the CAD patients were partitioned to the presence or absence of CAC, no association was found in either subgroup as well (Table 3).

**Table 3:** The association analysis for number of affected vessels using rs1333049 as predictors in a linear regression model in CAD patients and subgroups.

	Number of affected vessels (1-4)	
Population	β (95%CI)	*P*
All CAD patients	0.02(−0.10 to 0.14)	0.751
CAD patients without CAC	0.03(−0.13 to 0.20)	0.714
CAD patients with CAC	−0.02 (−0.20 to 0.16)	0.821

The correlation was shown with adjustment for age, sex, SBP, DBP, BMI, TC, TG, HDL-C, LDL-C, glucose, smoking, DM, and hypertension status. The rs1333049 GG genotype was as reference.

### rs1333049 was not associated with CAC severity or extent in CAD patients

The correlation between rs1333049 and CAC severity/extent in CAD patients was determined by analyzing rs1333049 and ln(CAC score+1) and the number of coronary calcified vessels both in subjects with simple CAC and in CAD patients. rs1333049 was correlated with the 2 indices of CAC in the whole CAD patient group, including patients with CAC and patients without CAC; however, in the subgroup analysis, the correlation disappeared in the subgroup with CAC (Table 4).

**Table 4:** The association analyses for ln(CAC score+1) and number of calcified vessels using rs1333049 as predictors in a linear regression model in all CAD patients and subgroup with CAC.

	Ln(CAC score+1)		Number of Calcified vessels (1–4)	
Population	β (95%CI)	*P*	β (95%CI)	*P*
All CAD patients	0.26 (0.04 to 0.48)	0.019	0.14 (0.02 to 0.26)	0.021
CAD patients with CAC	0.02 (−0.06 to 0.10)	0.625	0.06 (−0.08 to 0.20)	0.402

The correlation was shown with adjustment for age, sex, SBP, DBP, BMI, TC, TG, HDL-C, LDL-C, glucose, smoking, DM, and hypertension status. The rs1333049 GG genotype was as reference.

### The rs1333049 was not associated with the incidence of combined CV events

The clinical characteristics of the cohort study population are shown in Table 5. In these patients, 5 died of non-cardiovascular diseases and 7 died of cardiovascular disease. In the 241 subjects with CV events, the affected vessels were also recorded (as shown in Table 6). rs1333049 was not associated with the incidence of CV events in the whole cohort or in the subgroups with or without CAC (Table 7).

**Table 5:** Characteristics of patients enrolled in the cohort study grouped by CAC status and rs1333049 genotype.

rs1333049 genotype	GG	GC	CC
Number	182	413	254
Age, y	56.8(9.7)	57.9(9.5)	56.8(9.2)
Men, %	81.9	78.5	81.9
SBP, mmHg	125.1(14.3)	124.4(15.1)	123.7(13.7)
DBP, mmHg	78.3(9.3)	77.6(9.3)	76.0(9.3)*
BMI, kg/m^2^	25.9(3.1)	25.9(3.2)	26.2(3.0)
TG, mmol/L	1.62(7.43)	1.62(8.81)	1.63(10.07)
TC, mmol/L	4.64(1.19)	4.59(1.22)	4.59(1.13)
HDL-C, mmol/L	1.13(0.37)	1.12(0.34)	1.11(0.32)
LDL-C, mmol/L	2.53(0.96)	2.46(0.90)	2.47(0.86)
Glucose, mmol/L	5.91(1.44)	6.01(1.56)	6.07(1.66)
Cigarette smoking, %	69.8	61.5	65.7
Hypertension history, %	61.0	64.2	68.9
DM history, %	42.9	41.9	41.7
With CAC %	72.5	78.7	79.9
Ln(CAC score+1)	2.34(6.75)	2.46(8.05)	2.48(8.11)
DES/CABG	146/36	304/109	194/60

Age, BMI, SBP, DBP, glucose, HDL-C, LDL-C, and TC values are given as mean (SD); TG values and Ln(CAC score+1) were as median (range), and other values as percentages. *P<0.05 *vs* GG group

**Table 6:** Cardiovascular events in the cohort.

	Patients without CAC			Patients with CAC		
rs1333049 genotype	CC	CG	GG	CC	CG	GG
Patients N	51	88	50	203	325	132
Events in target vessels n(%)	13(25.5)	20(22.7)	10(20.0)	54(26.6)	79(24.3)	35(26.5)
Events in non-target vessels n(%)	7(13.7)	11(12.5)	7(14.0)	43(21.2)	47(14.5)	16(12.1)
Events n(%)	14(27.4)	24(27.3)	12(24.0)	63(31.0)	91(28.0)	37(28.0)

**Table 7:** The hazard ratio of combined events and events in non-target vessels for rs1333049.

		Hazard ratio(95% CI)	*P*
Combined events	Whole cohort	1.13(0.94–1.35)	0.191
	Subgroup with CAC	1.14(0.93–1.40)	0.213
	Subgroup without CAC	1.16(0.79–1.72)	0.444
Events in non-target vessels	Whole cohort	1.32(1.02–1.69)	0.031
	Subgroup with CAC	1.44(1.08–1.91)	0.012
	Subgroup without CAC	1.02(0.58–1.80)	0.934

The hazard ratio was adjusted with age, sex, SBP, DBP, BMI, TC, TG, HDL-C, LDL-C, glucose, smoking, DM, and hypertension status; GG group was as reference. In rs1333049, the allele coding was as the number of minor allele (C).

### rs1333049 was significantly associated with the incidence of CV events in non-target vessels in patients with CAC

To eliminate the interference of coronary revascularization, we also analyzed the association of rs1333049 with the incidence of CV events which occurred in non-target vessels. rs1333049 was significantly associated with the incidence of CV events which occurred in non-target vessels in patients with CAC, but not in patients without CAC (Table 7).

## Discussion

This is the first clinical investigation on the effects of variants at 9p21 locus on CAD subtypes in presence and in absence of CAC. We found that the rs1333049 allele increased the risk of CAD and CV events in the presence, but not in the absence, of CAC. We also found that the variant was not associated with simple CAC or with the extent or severity of CAC in CAD patients; therefore, calcification may be involved in the mechanism by which the 9p21 locus affects the susceptibility to CAD.

CAC is thought to be a precursor to, or complication of, CAD. Some have postulated that calcified plaques may represent “older, healed” plaques, as compared to calcified components within a mixed calcified plaque which have been proposed as intermediary in age between non-calcified and calcified plaques; however, this assertion, to date, has not been proven [22]. Thus far, many studies have indicated that arterial calcification is a complex, organized, and regulated process similar to bone formation, and that there is no particular reason why it should be a reliable indicator of either the plaque burden or the risk of a future CV event. In a recent publication, Nicoll et al. in 2013 reviewed previous studies on CAC and CAD and concluded that it is time to divorce arterial calcification from atherosclerosis and instead view it as a distinct pathology in its own right, albeit one which frequently coexists with atherosclerosis and is related to it for reasons which are not yet fully understood [23]. There is also a close relationship between CAC presence and atherosclerotic plaque burden, with angiography studies showing very high sensitivity but poor specificity of CAC score for predicting obstructive disease. Nevertheless, histopathology studies indicate that heavily calcified plaque is unlikely to result in a CV event, while the vulnerable plaque tends to be uncalcified or “mixed”, suggesting that calcification may be protective.

Different mechanisms were involved in the two CAD subtypes, in the absence and presence of CAC. Hyperlipidemia may be associated with the extent of non-calcified plaques [24]. Calcification in the arterial vasculature has been proposed to occur at the later stage of the atherosclerosis process [25]. Increased production and irreversible cross-linking of collagen fibers, reduced levels of elastin, infiltration of vascular smooth muscle cells, macrophages and mononuclear cells, and increased matrix metalloproteinases are the sequelae of vascular inflammation, which contribute to reduced arterial distensibility [26]. Siegel et al. in 2013 found that thoracic aortic distensibility was reduced locally at the site of thoracic atherosclerosis, specifically at sites of calcified and mixed thoracic aortic plaques, as compared regions of noncalcified plaques [27]. Furthermore, mortality is consistently higher in renal patients with CAC, with an even faster progression among those on haemodialysis [28], [29], with CAC development beginning at an earlier age [30]. Recent findings of associations of the same 9p21 locus with abdominal aortic and intracranial aneurysms [9] imply that this susceptibility gene affects the basic physiological properties of the arterial wall.

As a degenerative and inflammatory disease of elderly patients, about 80% of abdominal aortic aneurysms show considerable wall calcification [31]. Studies on the association of the locus with CAC [32]–[34] have not investigated the difference in its association with CAD in the absence or presence of CAC; but these results suggest involvement in the formation of calcified atherosclerotic lesions in coronary arteries. Our findings, together with previous studies, suggest that variants at the 9p21 locus may exert more general effects on arterial wall function, which are involved in mural remodeling in CAD, calcification-related atherosclerosis, abdominal aortic aneurysms, and intracranial aneurysms.

It is interesting to note that variants at the 9p21 locus robustly predict angiographic CAD prevalence, independent of standard risk factors, but do not predict CAD extent or myocardial infarction [6], [35], [36]. Additionally, variants at the 9p21 locus are not associated with aneurysm growth rate or risk of rupture [9]. In our study, no association was found between rs1333049 and the extent of CAD, as assessed by the number of affected vessels. Furthermore, no associations were observed with the severity of CAC as assessed by ln(CAC score+1) or the extent of CAC, as assessed by the number of calcified vessels in CAD patients with CAC. It is possible that the locus may act at an early stage (as an initiator) of vascular calcification-accompanied atherosclerosis. Future studies should be conducted on animal models in order to confirm this hypothesis.

Another interesting finding was that this locus was only associated with the incidence of CV events which occurred in non-target vessels. Previous studies reported that this locus is not related to the clinical and angiographic outcomes after the placement of DES in coronary arteries [20]; therefore, rs1333049 might be associated with a younger age on onset of CAD [37], as well as the progression of coronary atherosclerosis and the probability of coronary artery revascularization in early-onset myocardial infarction[21]. Ardissino et al in 2011 proposed that this contradiction can be explained by interference from coronary revascularization[21]. Our findings also confirmed the positive association in non-interfered vessels in patients with CAC who underwent revascularization. The involvement of calcification in this association was confirmed by our cohort study.

There were some limitations in our study. At first, the phenotype CAD and CAC were only determined by CCTA. CCTA has been shown to be a sensitive and specific tool for the detection of significant coronary stenosis and has also allowed for the visualization and characterization of plaque subtypes [38]–[41]. Second, the possible population stratification could result in a spurious association between a marker and disease. We genotyped an additional 7 unlinked microsatellite markers and found no significant allele-frequency differences between controls and patients, indicating that there was no obvious evidence for genetic stratification in the populations. Third, the association of 9p21 locus with CAD has been validated by multiple groups worldwide, across racial and geographic boundaries, so our observations in these Chinese populations should be confirmed in other populations in the future.

In conclusion, the variant on the 9p21 locus was only associated with CAD and CV events in the presence of CAC. This locus may only confer risk of calcification related to coronary atherosclerosis.
